# Supplementary material for: Monocyte Distribution Width, Neutrophil-to-Lymphocyte Ratio, and Platelet-to-Lymphocyte Ratio Improves Early Prediction for Sepsis at the Emergency
Source: J Pers Med. 2021 Jul 28;11(8):732. doi: 10.3390/jpm11080732 (PMC8402196; doi:10.3390/jpm11080732)
Supplement: Supplementary file 1 [file jpm-11-00732-s001.zip › jpm-1297646-supplementary.pdf]

Supplemental Materials.

Supplemental Table S1. Etiologies of Microorganisms

Microorganisms	Number	Percentage (%)
**Gram-negative bacili**	**250**	**63.0**
Escherichia coli	144	36.3
Klebsiella pneumoniae ssp pneumoniae	27	6.8
Enterobacter cloacae complex	10	2.5
Proteus mirabilis	10	2.5
Pseudomonas aeruginosa	10	2.5
Bacteroides fragilis	6	1.5
Salmonella species	6	1.5
Klebsiella oxytoca	5	1.3
Aeromonas hydrophila	4	1.0
Acinetobacter baumannii/calcoaceticus complex	3	0.8
Citrobacter koseri	3	0.8
Serratia marcescens	3	0.8
Aeromonas caviae	2	0.5
Campylobacter coli	2	0.5
Enterobacter cloacae	2	0.5
Providencia rettgeri	2	0.5
Stenotrophomonas maltophilia	2	0.5
Acinetobacter baumannii	1	0.3
Bacteroides thetaiotaomicron	1	0.3
Campylobacter jejuni	1	0.3
Citrobacter freundii	1	0.3
Klebsiella pneumoniae	1	0.3
Klebsiella pneumoniae ssp ozaenae	1	0.3
Leptotrichia goodfelloeii	1	0.3
Morganella morganii	1	0.3
Ralstonia pickettii	1	0.3
**Gram-positive cocci**	134	33.8
Staphylococcus aureus	28	7.1
Staphylococcus capitis	20	5.0
Staphylococcus epidermidis	11	2.8
Staphylococcus hominis	11	2.8
Streptococcus dysgalactiae	8	2.0
Streptococcus agalactiae (Strep. group B)	7	1.8
Enterococcus faecalis	5	1.3
Streptococcus anginosus	5	1.3
Staphylococcus haemolyticus	4	1.0
Staphylococcus pettenkoferi	4	1.0
Enterococcus faecium	3	0.8
Enterococcus casseliflavus	2	0.5
Lactococcus garvieae	2	0.5
Micrococcus luteus	2	0.5
Micromonas micros	2	0.5

Staphylococcus lugdunensis	2	0.5
Staphylococcus saprophyticus	2	0.5
Streptococcus agalactiae	2	0.5
Streptococcus gallolyticus	2	0.5
Streptococcus pyogenes (Strep. group A)	2	0.5
Aerococcus viridans	1	0.3
Enterococcus hirae.	1	0.3
Staphylococcus caprae	1	0.3
Streptococcus constellatus	1	0.3
Streptococcus cristatus	1	0.3
Streptococcus dysgalactiae ssp equisimilis	1	0.3
Streptococcus gordonii	1	0.3
Streptococcus mitis	1	0.3
Streptococcus oralis	1	0.3
Streptococcus vestibularis	1	0.3
**Gram-positive bacilli**	**11**	**2.8%**
Corynebacterium aurimucosum	2	0.5
Lactobacillus rhamnosus	2	0.5
Arcanobacterium haemolyticum	1	0.25
Corynebacterium afermentans	1	0.25
Corynebacterium argentoratense	1	0.25
Corynebacterium minutissimum	1	0.25
Corynebacterium striatum	1	0.25
Erysipelothrix rhusiopathiae	1	0.25
Solobacterium moorei	1	0.25
**Yeast**	**2**	**2**
Candida glabrata	2	0.5

Supplemental Table S2. SIRS Score Before and After Propensity Score Matching

	**Before PS matching (*N* = 8698)**			**After PS matching (*N* =1480)**		
**Variables**	**Sepsis**	**Nonsepsis**	**P value**	**Sepsis**	**Nonsepsis**	**P value**
Number (*N*)	308	8390		296	1184	
Mean SIRS score	2.6 ± 0.7	1.1 ± 1.0	<0.001	2.5 ± 0.6	1.2 ± 1.0	<0.001
SIRS score ≥ 1	308 (100%)	5528 (65.9%)	<0.001	296 (100%)	843 (71.2%)	<0.001
SIRS score ≥ 2	308 (100%)	2540 (30.3%)	<0.001	296 (100%)	452 (38.2%)	<0.001
SIRS score ≥ 3	141(45.8%)	691 (8.2%)	<0.001	133 (44.9%)	154 (13.0%)	<0.001
SIRS score ≥ 4	27 (8.8%)	61 (0.7%)	<0.001	24 (8.1%)	19 (1.6%)	<0.001

Abbreviations: SIRS, Systemic Inflammatory Response Syndrome; N, number; PS, propensity score.

Supplemental Table S3. Diagnostics for SIRS Score Before and After Propensity Score Matching

	**Before PS matching (*N* = 8698)**		**After PS matching (*N* =1480)**	
**Variables**	**Sensitivity**	**Specificity**	**Sensitivity**	**Specificity**
SIRS score ≥ 1	100.0%	34.1%	100.0%	28.8%
SIRS score ≥ 2	100.0%	69.7 %	100.0%	61.8 %
SIRS score ≥ 3	45.8%	91.8%	44.9%	87.0%
SIRS score ≥ 4	8.8%	99.3%	8.1%	98.4%

Abbreviations: SIRS, Systemic Inflammatory Response Syndrome; N, number; PS, propensity score.
